# Professional Conduct in the Seventeenth Century

**Published:** 1912-06-08

**Authors:** Frederic Bullen


### MEDICAL ANTIQUITIES.

### Professional Conduct in the Seventeenth Century.

By FREDERIC BULLEN.

The following extracts are from the Statutes of the College of Physicians, which were published in 1693, rather more than 170 years after the College had been founded by Thomas Linacre, its first President. As a testimony to the antiquity of professional ethics they are not without interest:—

"No Physician who shall be called second to a sick Person shall cause the former Physician to be repelled or put by; nor shall he alter any Thing unless the Matter be urgent, before he meet his Colleague; and that there be no place for Deceit, whoever is sent for to a sick Person, shall enquire of him, or of the By-standers, whether anyone hath prescribed any Medicine, under a Penalty.

### Consultations and Visits.

"If to visit the same sick Person, two or more Physicians shall meet together, let none prescribe any Thing, nay, let him not so much as hint what is to be done in the presence of the Sick, or By-standers, before, with joint Council in Private, it shall be concluded betwixt them; lest he seem too ambitiously to forestal Practice, and snatch the free Opportunity of prescribing from all the rest; unless by some sudden and urgent Occasion, and that to be approved by the President and Censors, he shall be compelled to prescribe alone, under a Penalty.

"Lest any Strife or Controversy should arise between Physicians for officious Visitings, Fore-stalments, and Insinuations, we appoint and ordain, that when other Physicians shall be called to any Physician to consult, then the elder Physician, or some other relate to the Sick or By-standers what, by common Consent has been approved and prescribed; and let the rest leave the Execution thereof to the ordinary Physician. Nor shall they again visit the sick, unless expressly desired to do so by the ordinary Physician, or the sick Person, under a Penalty.

"No colleague shall print, divulge, or by any means publish any Book, Treatise, Table, or any Paper or Pamphlet whatsoever wherein is contained any Thing that relates to the Art of Physic or Surgery, or the Practice of them without Leave and Approbation of the President and Censors, or the greater Part of them. Offenders against this and the above Laws are at first to be fined, and afterwards expelled.

"If by chance any Surgeon or Apothecary shall offer himself to the President and Censors to be examined that he may be received into the Number of Candidates or Licentiates; we will that he, before Examination, or at least before Admission, be wholly discharged from all Tie and Obligation, wherewith he was formerly obliged to his Community."

From the same Statutes we learn that Fellows of the College were previously limited to forty in number, but under a Charter from King James the Second that number was extended to eighty. "They must have been one whole Year Candidates, or have publicly read Physic in some University of *England*, or have been Doctors of the Chair (as they say) in some University of this Kingdom; or the King's Physician in Ordinary."

The "candidates" here referred to were twelve in number, must not be foreigners, and must have practised physic for four years prior to being admitted into the order.

For foreign practitioners there was an Honorary Fellowship, which was also given to those who for some reason were not available as candidates. They were allowed to practise in London and for seven miles round, but had no votes in the affairs of the College. Finally, there were the "Licentiates," who were, it is hoped, extremely modest men, because they are described as "such other Persons skilled in Physic; who, by Reason of their being Foreigners or their not being admitted Doctors in one of our Universities, or for their not being eminently learned, or by reason of their too great Youth, or such like Causes, are not capable to be elected into the Number of Candidates; yet may, notwithstanding, be serviceable to the Public in taking care of the Health of the King's Subjects, at least in some particular Diseases: these are suffered to practise in London and seven miles round."

### The Form of Examination.

For admission to the Fellowship or Candidature the following was the form of examination:—

"First, let him be examined in the *Physiologic* Part, and the very Rudiments of Medicine; and in the Examination let Questions be propounded out of Books concerning Elements, Temperaments, the use of Parts, Anatomy, natural Powers and Faculties, and the other parts of Natural Medicine. Secondly, let him be examined in the *Pathologic* Part, or concerning the Causes, Differences, Symptoms, and Signs of Diseases which Physicians make use of to know the Essence of Diseases; and in his Examination, let Questions be proposed out of Books concerning the Art of Physic, of the places affected, of the Differences of Diseases, and Symptoms of Fevers, of the Pulses, of the Books of Prognostics of *Hyppocrates*, etc. Thirdly, let him be examined concerning the Use and Exercise of Medicine, or the Reason of Healing; and let that be done out of the Books concerning Preservation of Health, of the Method of Healing, of the Reason of Diet in acute Diseases, of Simple Medicines, of Crises, of the Aphorisms of *Hyppocrates*, and other Things of that kind which relate to the use of Healing: For example sake, What Caution is to be observed in purging? what in Blood-letting? at what Time? in what Disease? in what Person? with what Medicine? And in what Vein those Things ought to be done? Likewise, what is the Use of

*Narcotics*, and Sleeping Medicines? what Caution is to be observed in them? What is the Position and Site of the internal Places? And by what Passages Medicines come to them? What is the use of Glysters? What of Vomits? The Danger, Kind and Measure?" A syllabus happy, surely, for its brevity, if not for its completeness.

The duties of the censors, referred to in the former part, included the difficult tasks of searching the apothecaries' shops, to judge their medicines and to burn or destroy the corrupt; they were to enquire after all persons practising physic in London and to "examine, correct and govern them, to enquire their methods of cure and if need be of a Law-suit, together with the President and Treasurer to persecute." There were four censors appointed, with these great powers of visitation and prosecution, and the insufficiency of these caused Sir Theodore Mayerne and Henry Atkins, Physicians to King James the First, to take steps to form the apothecaries into a distinct society, by means of another charter, with powers of visitation, and expressly prohibiting all persons from exercising the business of an apothecary within seven miles of the metropolis unless first licensed by the Company of Apothecaries.

